# Effects of serum on cytotoxicity of nano- and micro-sized ZnO particles

**DOI:** 10.1007/s11051-013-1829-5

**Published:** 2013-08-27

**Authors:** I-Lun Hsiao, Yuh-Jeen Huang

**Affiliations:** Department of Biomedical Engineering and Environmental Sciences, National Tsing Hua University, No. 101, Section 2, Kuang-Fu Road, Hsinchu, 30013 Taiwan, ROC

**Keywords:** Nanoparticles, Cytotoxicity, A549, Zinc oxide, Serum

## Abstract

Although an increasing number of in vitro studies are being published regarding the cytotoxicity of nanomaterials, the components of the media for toxicity assays have often varied according to the needs of the scientists. Our aim for this study was to evaluate the influence of serum—in this case, fetal bovine serum—in a cell culture medium on the toxicity of nano-sized (50–70 nm) and micro-sized (<1 μm) ZnO on human lung epithelial cells (A549). The nano- and micro-sized ZnO both exhibited their highest toxicity when exposed to serum-free media, in contrast to exposure in media containing 5 or 10 % serum. This mainly comes not only from the fact that ZnO particles in the serum-free media have a higher dosage-per-cell ratio, which results from large aggregates of particles, rapid sedimentation, absence of protein protection, and lower cell growth rate, but also that extracellular Zn^2+^ release contributes to cytotoxicity. Although more extracellular Zn^2+^ release was observed in serum-containing media, it did not contribute to nano-ZnO cytotoxicity. Furthermore, non-dissolved particles underwent size-dependent particle agglomeration, resulting in size-dependent toxicity in both serum-containing and serum-free media. A low correlation between cytotoxicity and inflammation endpoints in the serum-free medium suggested that some signaling pathways were changed or induced. Since cell growth, transcription behavior for protein production, and physicochemical properties of ZnO particles all were altered in serum-free media, we recommend the use of a serum-containing medium when evaluating the cytotoxicity of NPs.

**Electronic supplementary material:**

The online version of this article (doi:10.1007/s11051-013-1829-5) contains supplementary material, which is available to authorized users.

## Introduction

As new nanomaterials are increasingly synthesized and applied in consumer products, occupational workers and end-product users are more likely to become exposed to potentially harmful effects of these substances. However, progress in the risk and health assessment of these new materials lags. In recent years, researchers have begun to investigate the adverse effects of nanoparticles (NPs) and their mechanisms (Oberdorster et al. 2005; Casals et al. 2008; Xia et al. 2008; Hsiao and Huang 2011a, c). Among all nano-safety issues, the effect of physicochemical properties of NPs on biological responses is widely studied (Rivera Gil et al. 2010; Shvedova et al. 2010; Hsiao and Huang 2011a). However, optimal exposure conditions, such as exposure duration for biological in vitro and in vivo studies has not been standardized, nor has an NP dispersion guide been proposed. This situation has led to some arguments over how to determine the effects of the physicochemical characteristics (e.g., size, shape, surface area, and surface chemistry) of NPs on their biological responses (Murdock et al. 2008).

When discussing experimental conditions for in vitro studies, the distinction between using serum-containing or serum-free media for biological assays is one of important issues (Jones and Grainger 2009). Although many studies have used serum-containing media for NP-exposure studies (Karlsson et al. 2008, 2009; Vamanu et al. 2008; Choi et al. 2009), others have used serum-free media without explaining why they chose to avoid serum (Brown et al. 2007; Chen et al. 2008; Braydich-Stolle et al. 2009; Yuan et al. 2010). Furthermore, the amount of serum used in cytotoxicity studies sometimes differs in studies of the same cell line—with 5 or 10 % serum commonly used in the medium.

Serum, which contains essential proteins and nutrients for the in vitro system, is commonly used as a supplement in cell culture media. Serum can also be a stabilizing agent for NPs (Murdock et al. 2008; Allouni et al. 2009; Cronholm et al. 2011). In view of the potential discrepancies, a few groups have begun to evaluate the effects of serum on particle toxicity from the aspect of particle agglomeration and surface chemistry (Davoren et al. 2007; Murdock et al. 2008; Zhu et al. 2009). For example, the presence of protein-containing serum in a medium has a protective effect on the toxicity of both silica and single-walled carbon nanotubes (Schimmelpfeng et al. 1992; Davoren et al. 2007). Recently, studies have found that the protective effect on NP toxicity was derived from the binding of multiple layers of serum proteins (e.g., bovine fibrinogenon, gamma globulin, etc.) on NPs to protect cells from direct interaction (Ge et al. 2011). These “protein corona” not only change cellular responses, but also determine kinetics and distribution of NPs in vivo (Walkey and Chan 2012). Different kinds of protein corona adsorbed onto silica NPs in the presence or absence of serum, and even altered adhesion of particles to the cell surface, affect uptake, toxicity, and particle localization (Lesniak et al. 2012).

Nano-ZnO has become an attractive nanomaterial for use in photochemical and biological fields (Yang and Chan 2009; Kumar and Chen 2008). Many studies have demonstrated, however, that nano-ZnO has higher cytotoxicity than those of other metal oxide or carbon-based NPs (Lin et al. 2009; Yang et al. 2009). Nano-ZnO also induces toxicity in various mammalian cells (human BEAS-2B, A431, A549 and mouse C17.2), leading to potent DNA damage, inflammation, and even cell apoptosis (Huang et al. 2010; Deng et al. 2009; Sharma et al. 2009; Lin et al. 2009). Due to its high toxicity, some studies also dedicated to remedy it by iron doping or TiO_2_ coating (Xia et al. 2011; Hsiao and Huang 2011c). The cytotoxicity of nano-ZnO appears to be associated with the release of zinc ions (Zn^2+^), suggesting the absence of size-dependent toxicity as the dosage falls lower than 18.2 μg/mL (Xia et al. 2008; Deng et al. 2009); nevertheless, some studies indicate particle–cell contact which induces reactive oxygen species, leading to cytotoxicity (Yang et al. 2009; Moos et al. 2010). In the absence of serum, nano-ZnO exposed to A549 cells caused a significant decrease in the cytokinesis-block proliferation index (CBPI) at 25 μg/mL, while in the presence of 10 % serum the same result showed at only 50 μg/mL (Corradi et al. 2012). The nano-bio-complex, with selective bindings of components or ions of medium to nano-ZnO, could be observed in Dulbecco’s modified Eagle’s medium (DMEM) w/wo serum and phosphate-buffered saline (PBS). The bindings were found to be independent of serum proteins (Xu et al. 2012). In summary, the complexity of serum on the sample characters, solution properties and toxicity of ZnO particles have not been well understood.

In this study, we investigated the influence of serum in the DMEM medium on the toxicity of nano- and micro-sized ZnO toward human lung epithelial cells. We wanted to understand and explain: (1) if there are any differences in the results of cytotoxicity on different sizes of ZnO particles, (2) what is the extent of extracellular Zn^2+^ released from ZnO suspension and its role on the cytotoxicity and, (3) if the correlation of endpoints (cytotoxicity and inflammation) changes in various serum conditions. We discuss our biological response data (mitochondria activities and production of pro-inflammatory factor IL-8) under different exposure conditions of ZnO particles by varying the size distribution, hydrodynamic size, sedimentation states, and zinc ion release. Herein, appropriate medium components for in vitro studies of NPs are suggested.

## Materials and methods

### Chemicals

Nano-sized (50–70 nm) and micro-sized (<1 μm) ZnO commercial powders were purchased, respectively, from Sigma-Aldrich Corp. (St. Louis, MO, USA) and Sun Beam Tech (Taiwan). All aqueous solutions used in the chemical and biological experiments were prepared from 18.2 MΩ cm ultrapure de-ionized and sterilized water, obtained from Millipore Simplicity Systems (Millipore, S.A.S.).

### Size and surface area analysis

The particle sizes and morphologies of the commercial powder were analyzed using a TECNAI 20 transmission electron microscope (Philips, Netherlands). To prepare the samples, NPs were first dispersed in absolute ethanol (100 μg/mL) and then sonicated (400 W, 40 kHz) for 30 min. Aliquots (4 μL) of the suspensions were deposited onto 200-mesh carbon-formvar Cu grids. Finally, the solvents were evaporated in a vacuum oven for 1 h. The surface area of ZnO particles was determined by N_2_ adsorption–desorption isotherms obtained at 77 K using ASAP 2020 Micromeritics Surface Area and Porosity Analyzer. Surface area was calculated by the Brunauer–Emmett–Teller (BET) method.

### Size distribution measurements in air and medium system

To determine the real-size distributions of the ZnO particles, an electrical low-pressure impactor (ELPI, Dekati, Finland) was used to monitor the size distribution of the particles in air. The ELPI could measure the airborne particle size distribution in the size range of 0.03–10 μm with 12 channels. First, an amount of powder (after storage in a drying box for 1 week) was placed in a self-made chamber (length 0.5 m; width 0.4 m; height 0.5 m). Using a blower (1.2 m^3^/min), the particles were agitated for 30 min and collected by the ELPI. The alumina foils in each channel sensed the charged particles and a program calculated its number distribution. The temperature during measurement was 16.3 °C; the relative humidity was 75 %.

The hydrodynamic size and surface charge of the ZnO particles in water and the cell culture medium were monitored using a Zetasizer Nano ZS apparatus (Malvern Instruments, UK). The hydrodynamic size of the particles is expressed herein as the Z-average size, which was derived from the cumulant mean of the intensity autocorrelation function in the Zetasizer system. The number distribution of the particles in the medium was obtained through conversion of the volume distribution (Malvern Instruments 2004). Prior to measurement, samples were dispersed (at 100 μg/mL) in water and in DMEM (DMEM High Glucose, w/l-Glutamine, Gibco, USA) supplemented with 0, 5, or 10 % fetal bovine serum (FBS; Gibco, USA). To decrease the agglomeration state, the dispersions were sonicated in an ice bath using an ultrasonic probe (7 W, 22 kHz) for 7 min prior to measurement.

### Sedimentation study

The sedimentation rates of the particles were analyzed by monitoring the optical absorbance (at 367 and 378 nm for the nano- and micro-sized ZnO, respectively) as a function of time, within 24 h, using UV–Vis spectrophotometry (Varian 50 Bio, CA, USA). The dispersion guide for all suspensions was the same as that for the hydrodynamic size measurements described above.

### Analysis of zinc ions in medium

The levels of Zn^2+^ release in the serum-containing and serum-free media were compared for the nano- and micro-ZnO. Particles from a stock suspension (2 mg/mL) were diluted to 40, 20, and 10 μg/mL with serum-free DMEM and DMEM/10 % FBS, then incubated for 24 h at 37 °C. Aliquots (1 mL) were then centrifuged (15,000×*g*, 25 min) and diluted (1:40, v/v) with water. The resulting zinc solutions were analyzed using inductively coupled plasma mass spectrometry (ICP-MS, Perkin-Elmer SCIEX ELAN 5000). The Zn^2+^ ion concentration was determined from the intensity of the Zn isotope having a mass-to-charge (*m*/*z*) ratio of 66; an external standard curve was used.

### Cell culture

The human lung carcinoma epithelial cell line A549 (BCRC-60074, Hsin-Chu, Taiwan) was cultured in DMEM supplemented with 10 % FBS and 1 % Antibiotic–Antimycotic and then cultivated in T25 flasks at 37 °C in a humidified atmosphere of 5 % CO_2_/95 % air.

### Sample preparation

To prepare the stock solution, ZnO powders were suspended in sterile water at a concentration of 2 mg/mL. Prior to the next step of dilution, they were dispersed using a 5-W probe sonicator (Ultrasonic Cell Disruptor, Misonix, USA) for 1.5 min in an ice bath. Stock solutions were then diluted to concentrations ranging from 80 to 5 μg/mL in DMEM supplemented with 0, 5, or 10 % FBS. All diluted suspensions were then sonicated using a sonicator bath for 10 min at 400 W to break up the agglomerates. Finally, the cells were treated with these suspensions. In order to evaluate the effect of Zn^2+^ release from ZnO particles on cytotoxicity, the supernatants of ZnO particles from 10, 20, and 40 μg/mL of ZnO suspensions were used to treat cells (in DMEM supplemented with 0 or 10 % FBS). After preparing the ZnO suspensions, they were incubated for 24 h at 37 °C and centrifuged at 15,000×*g* for 25 min. The supernatants then could be obtained. For assessment of the cytotoxicity, the cells (4 × 10^4^ cells/mL) were seeded in 96-well plates and then exposed to ZnO suspensions or supernatants for 24 h after 24 h of cell attachment.

### Cell morphology

Cell morphology was assessed using an inverted optical microscope (Zoomkop EZ-20I, Leader Scientific) coupled to a digital compact camera system (SP-350, Olympus).

### Mitochondria activity

After exposure, the cell medium was aspired and the aliquots were transferred to a clean flat-bottomed plate for subsequent IL-8 analysis. Cells were washed once with 1× phosphate-buffered saline to remove residual NPs. Next, 200 μL of a 3-(4,5-dimethylthiazol-2-yl)-2,5-diphenyltetrazolium bromide (MTT)/DMEM (with 10 % FBS) solution (0.5 mg/mL) was added to each well; the plate was then incubated for 3 h. After incubation, the MTT solution was discarded and DMSO (200 μL) was added. Finally, the absorbance was measured at 570 nm using a tunable microplate reader (VersaMax, Molecular Devices, USA). The MTT activity (%) was calculated using the expression ${{\left[ {\text{cell number of testing samples}} \right]}/{\left[ {\text{cell number of control}} \right]}} \times 100.$

### Production of pro-inflammatory factor

A human IL-8 enzyme-linked immunosorbent assay (Human IL-8 ELISA Kit and Buffer, PeproTech, USA) was used to evaluate the production of the pro-inflammation chemokine IL-8. After a 24 h treatment, supernatants in each well were collected and analyzed according to the manufacturer’s protocol.

### Statistics

One-way analyses of variance (ANOVA) followed by Dunnett’s multiple comparison tests and two-tailed Student’s *t* test were used, respectively, to evaluate for the significance between control and each of the other samples, as well as to compare cytotoxic differences between nano- and micro-sized ZnO. A subsequent two-way ANOVA was applied to determine for the respective effects after adjustment for serum and particle size categories. Linear regression analysis was used to evaluate the correlation level among the cytotoxicity and inflammation responses.

## Results

### Characterization of ZnO particles

**Fig. 1:**
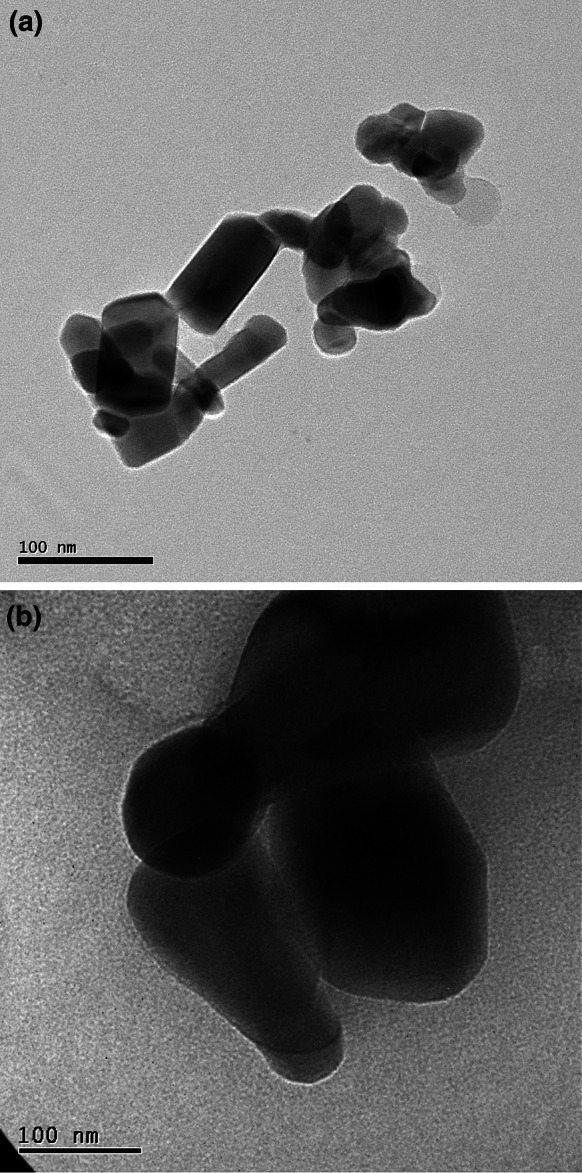
TEM images of ZnO particles. **a** nano-ZnO. **b** micro-ZnO

**Fig. 2:**
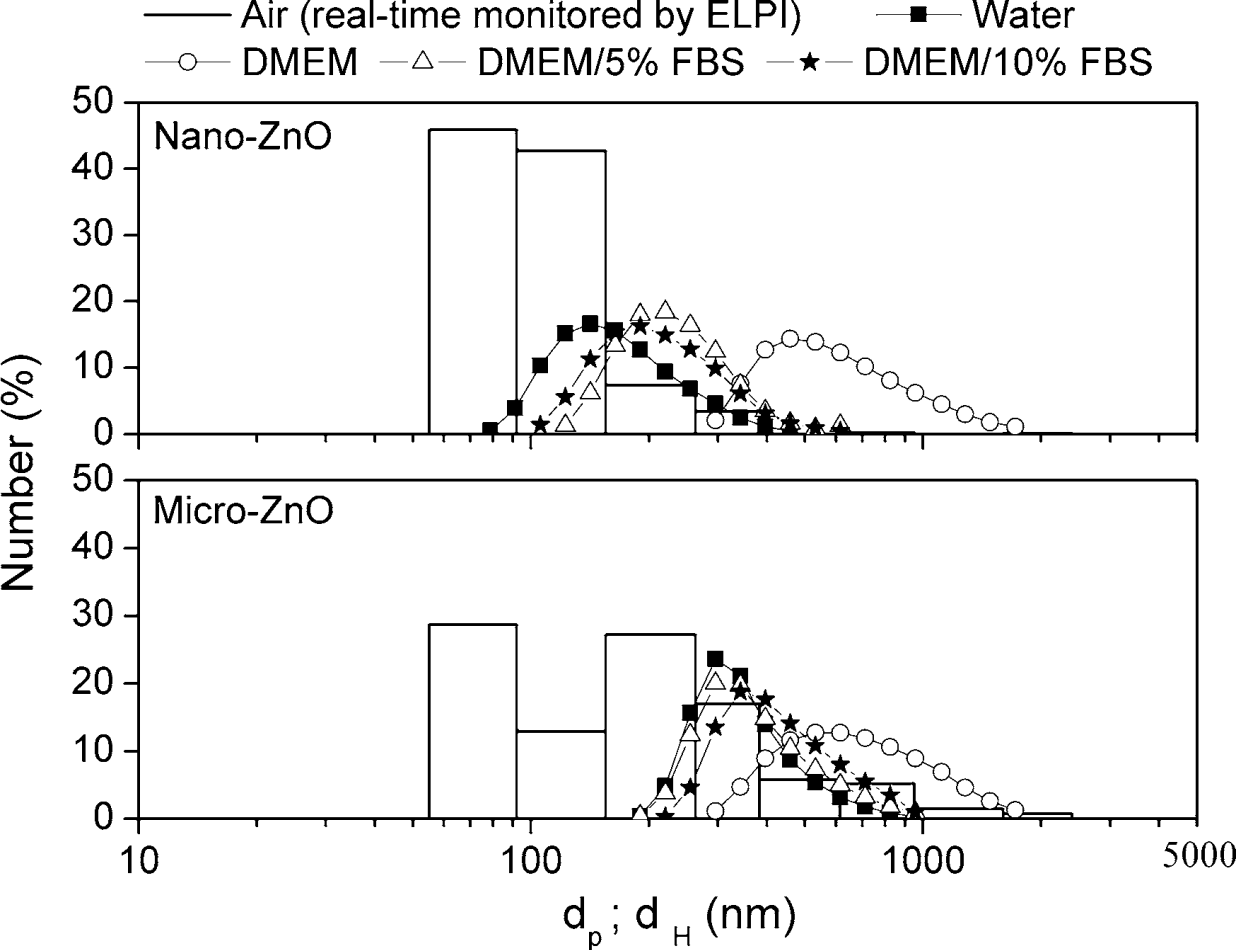
Size distributions of ZnO particles suspended in air, water, and DMEM with various percentages of FBS (*d*_p_ aerodynamic diameter; *d*_H_ hydrodynamic diameter)

Figure 1 presents TEM images of the nano- and micro-sized ZnO. Both commercial nanopowders aggregated slightly in ethanol, and their morphologies were non-uniform. The primary size corresponded to our expectation (nano-size ZnO: 50–70 nm; micro-size ZnO: <1 μm). To assess the differences in size distributions between the powder forms and medium suspensions, we measured the size distributions for nano- and micro ZnO in air and in aqueous medium (Fig. 2). From the ELPI real-time monitoring data, the size distribution of the ZnO NPs, based on the aerodynamic diameter, was 55–280 nm, although almost all of the particles had a diameter of approximately 100 nm. The size distribution of micro-sized ZnO mainly ranged from 55 to 1,000 nm, with only a small portion being described as having nanoscale dimensions. The specific surface area (SSA) of nano- and micro ZnO was 7.34 and 1.81 m^2^/g, respectively.

When ZnO NPs were dispersed in de-ionized water, they aggregated slightly to 279 nm (Table 1); in contrast, the aggregation of the micro-ZnO was less serious (530 nm). When the nano- and micro-sized ZnO particles were suspended in serum-free DMEM, their sizes increased to 936 and 1,107 nm, respectively. The agglomeration of nano- and micro-sized ZnO decreased when FBS was present in the cell culture medium. The aggregate sizes of the nano-ZnO in DMEM containing 5 and 10 % FBS were 320 and 329 nm, respectively; those of the micro-sized ZnO in DMEM containing 5 and 10 % FBS were 554 and 634 nm, respectively. Nevertheless, neither DMEM/5 % FBS nor DMEM/10 % FBS caused the size distribution of the ZnO particles to come close to their aerodynamic sizes. In addition to the mean size information, cumulant analysis also provided a polydispersity index (PDI), a width parameter. From the zeta potential analysis (Table 1), the two kinds of ZnO particles both featured positive surface charge in de-ionized water (22–26 mV); after suspension in DMEM medium, the zeta potentials turned negative (−2.4 and −4.0 mV for nano- and micro-ZnO, respectively) and became close to the zero-point of charge (ZPC). The zeta potentials of the ZnO particles decreased slightly, reaching between −7 and −10 mV, in DMEM/5 % FBS and DMEM/10 % FBS.

**Table 1:** Hydrodynamic sizes (HDS) and zeta potentials (Zp) of nano- and micro-sized ZnO in media containing different serum contents

Sample	Primary size (nm)	Surface area (m^2^/g)	HDS (nm) and Zp (eV)							
			Water		DMEM		DMEM/5 % FBS		DMEM/10 % FBS	
			HDS	Zp	HDS	Zp	HDS	Zp	HDS	Zp
			PDI		PDI		PDI		PDI	
Nano-ZnO	50–70	7.34	279 0.18	25.9	936 0.18	−2.4	320 0.31	−7.5	329 0.17	−9.3
Micro-ZnO	<1,000	1.81	530 0.21	22.1	1107 0.35	−4.0	554 0.23	−8.2	634 0.28	−7.8

### Sedimentation study

**Fig. 3:**
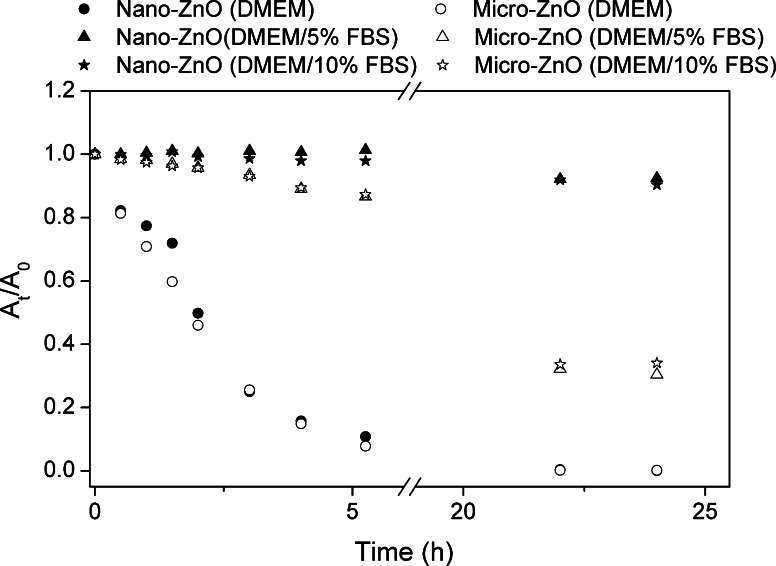
Sedimentation *curves* of ZnO particles (100 μg/mL) for various percentages of FBS in DMEM, *y*-axis expressed in terms of absorbance at time *t* (*A*_*t*_) relative to that at *t* = 0 (*A*_0_)

Figure 3 displays the sedimentation state of the nano- and micro-sized ZnO in the various media. Particles in serum-free DMEM exhibited a faster sedimentation rate than those in the serum-containing media. Almost all of the particles (both nano- and micro-ZnO) precipitated within 24 h; that is, they were very unstable in serum-free media. In contrast, the sedimentation rates were slower when FBS was present. The same particles had similar stabilities in the various FBS-containing media. Nevertheless, the micro-sized ZnO underwent faster sedimentation than did the nano-sized ZnO in both FBS-containing media.

### Cell morphology

**Fig. 4:**
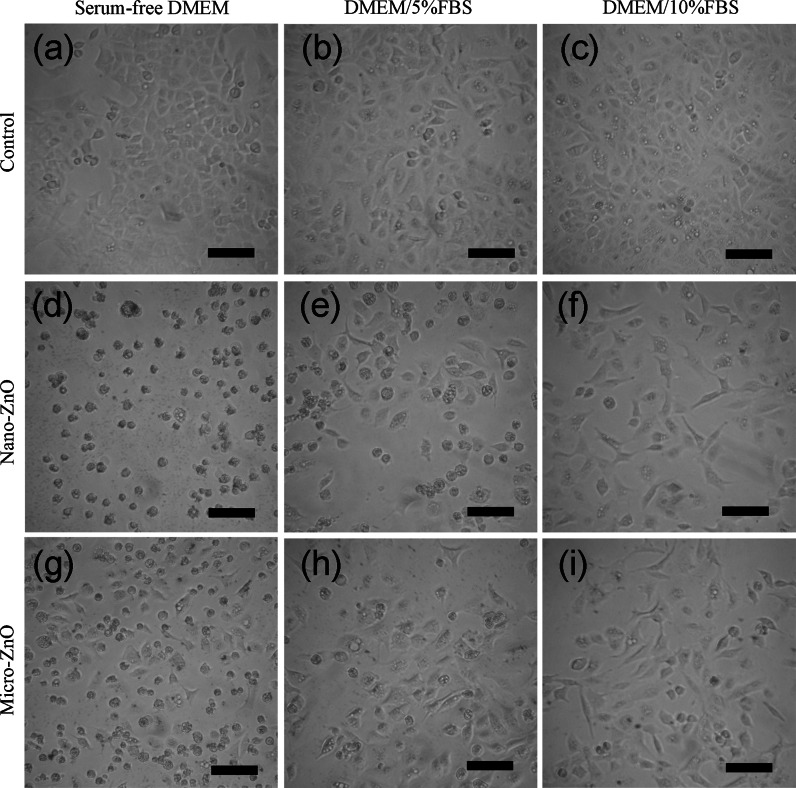
Morphologies of A549 cells exposed to 20 μg/mL of nano- and micro-ZnO for 24 h in DMEM with various percentages of FBS. **a**–**c** Cell control groups. **d**–**f** Cells treated with nano-ZnO. **g**–**i** Cells treated with micro-ZnO. The *scale bar* is 100 μm

The morphologies of the A549 cells grown without serum and with 5 or 10 % serum (control group) in the DMEM medium were not significantly different after 24 h (Fig. 4a–c). There was, however, the highest confluence and density of cells cultured in the medium incorporating 10 % FBS. When the A549 cells were treated with 20 μg/mL of nano-ZnO in serum-free medium, almost all of the cells retracted into a partially smaller spherical shape and exhibited decreased confluence from those in control (Fig. 4d); a smaller portion of the cells exhibited the same changes in morphology at the same concentration of the suspensions in the serum-containing medium (Fig. 4e, f). In the 20 μg/mL of micro-ZnO treated group, some cells with normal morphologies could be observed in serum-free medium (Fig. 4g). A small portion of cells exhibited abnormal changes in morphology at the same concentration of the suspensions in the serum-containing medium (Fig. 4h, i).

### Cell viability of ZnO particles

**Fig. 5:**
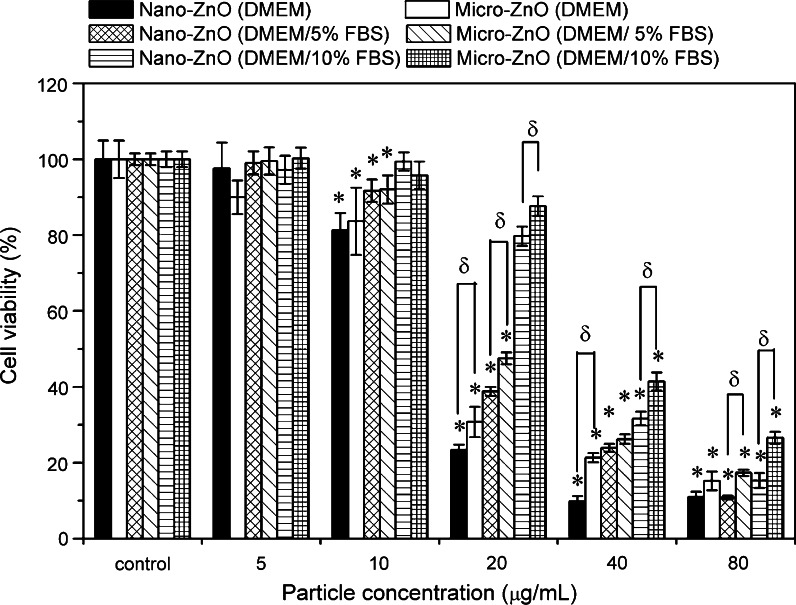
MTT activities of A549 cells after 24 h exposure to nano- and micro-ZnO in DMEM with various percentages of FBS. *p < 0.05 compared to untreated controls; ^δ^ *p* < 0.05 compared between treated groups. Particle size and serum-related contrasts were both observed by two-way ANOVA, *p* < 0.001 at 20–80 μg/mL

Figure 5 presents A549 cell viability of the nano- and micro-ZnO in DMEM supplemented with 0, 5, and 10 % FBS. The average optical densities of the MTT formazan in the 0, 5, and 10 % FBS/DMEM control groups were 1.4, 2.5, and 2.8, respectively, revealing that the rate of cell growth was proportional to the amount of serum added. Dose-dependent declines in viability occurred in each kind of medium. Two-way ANOVA revealed significant differences in toxicity for samples treated in three different medium conditions after adjustment for particle size (*p* < 0.001 at 10–80 μg/mL). The toxicities for both sizes of ZnO particles in the different media decreased in the following order: in serum-free DMEM > in DMEM/5 % FBS > DMEM/10 % FBS. Furthermore, in all medium conditions, significant differences in cytotoxicity were found when comparing nano-ZnO and micro-ZnO samples by Student’s *t* test (*p* < 0.05 at 20–80 μg/mL). The results indicated that regardless of the conditions, the nano-sized ZnO was more toxic than the micro-sized ZnO at concentrations of 20–80 μg/mL. However, the size-dependent effect could not be observed at concentrations of 5 and 10 μg/mL.

**Fig. 6:**
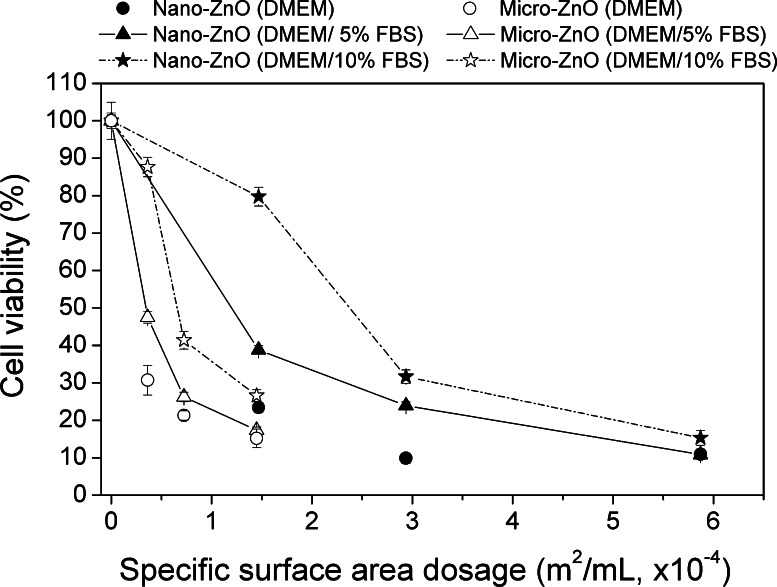
MTT activities of A549 cells after 24 h exposure to 20, 40, and 80 μg/mL of nano- and micro-ZnO using particle SSA as dosimetry

Mass concentration dosages in Fig. 5 were converted to SSA-based dosages in Fig. 6. In the same SSA, micro-ZnO was more toxic than nano-ZnO in both serum-containing media (Fig. 6). At 1.4 × 10^−4^ m^2^/mL of dosage, the differences of cell viability between nano- and micro-ZnO in serum-free media became smaller than in serum-containing media. Moreover, the cell viability showed a single tendency of dose-dependence decline within both two materials in serum-free media.

### Release of zinc ions in media and its cytotoxicity

**Fig. 7:**
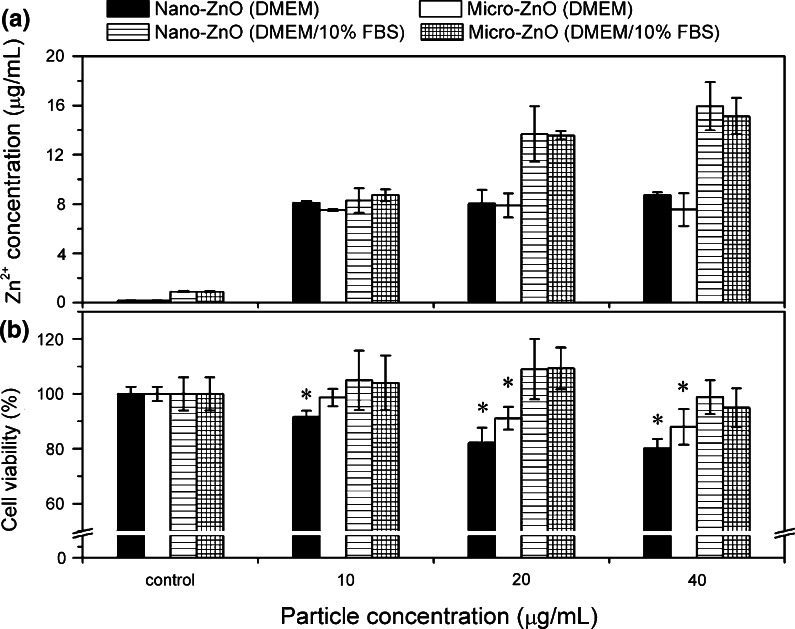
Dissolution of ZnO particles in cell medium and cytotoxicity for dissolved zinc. **a** Zn^2+^ released from nano- and micro-ZnO in serum-free DMEM and DMEM/10 % FBS after 24 h and 37 °C incubation. **b** MTT activities of A549 cells after 24 h exposure to supernatant of ZnO particles suspensions. *p < 0.05 compared to untreated controls

To assess the extent of Zn^2+^ released in the serum-containing and serum-free media, we evaluated the effects of the two ZnO particles at different concentrations (Fig. 7a). In the same medium and at the same concentration, we observed no significant differences in the release of Zn^2+^ between the two sizes of ZnO particles. Nevertheless, 40 μg/mL of nano-ZnO in DMEM/10 % FBS released 15.9 μg/mL of Zn^2+^ within 24 h, more than that in the serum-free medium (8.7 μg/mL). We observed a similar level in Zn^2+^ release in two different media for nano-ZnO at 10 μg/mL (8.3 μg/mL in DMEM/10 % FBS; 8.1 μg/mL in serum-free DMEM). On the other hand, the increase of Zn^2+^ could be seen as the concentration of the ZnO suspension increasing from 10 to 40 μg/mL in DMEM/10 % FBS, while there was a similar level of zinc ion release in serum-free medium. The morphologies of nano-ZnO in DMEM or DMEM/10 % FBS media are given in Supplementary Information.

To determine the Zn^2+^ release contributing to the cytotoxicity of ZnO particles in various media, we analyzed the cytotoxicity induced by supernatants of ZnO particle suspensions. As shown in Fig. 7b, there are no cytotoxicity differences between nano-ZnO and micro-ZnO-derived supernatants, both in serum-free and DMEM/10 % FBS. When the supernatants were derived from serum-free media, significant cytotoxicity could be observed markedly. However, when the supernatants were composed of DMEM/10 % FBS, there were no cytotoxicity for 10–40 μg/mL of ZnO supernatants.

### Pro-inflammatory factor responses and relation between cellular effects

**Fig. 8:**
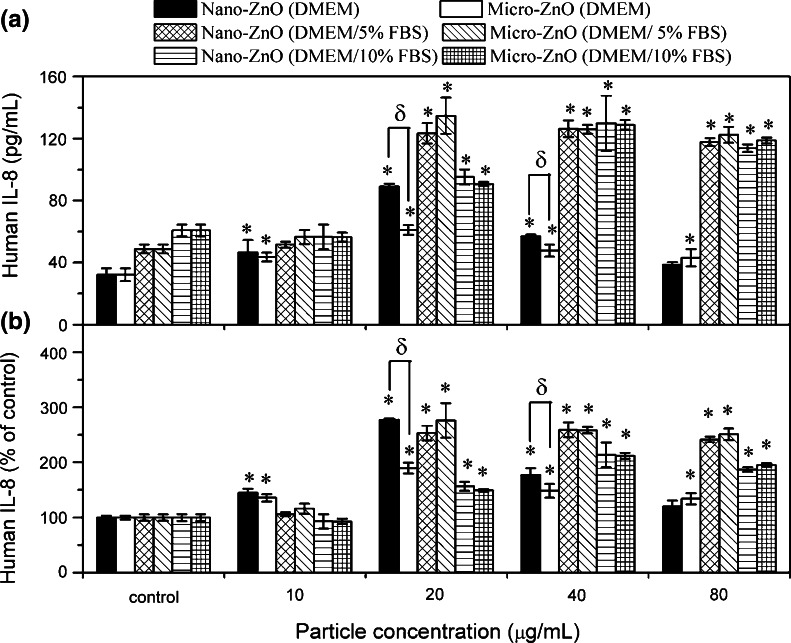
IL-8 production from A549 cells after 24 h exposure to nano- and micro-ZnO in DMEM with various percentages of FBS. **a** *y*-axis is expressed as production of IL-8 (pg/mL). **b** *y*-axis is expressed as IL-8 releasing folder (% of control). *p < 0.05 compared to untreated controls; ^δ^ *p* < 0.05 compared between treated groups. Two-way ANOVA analysis revealed significant contrasts related to serum factor for all dosages, upon adjustment of particle size, *p* < 0.01

Figure 8a shows the IL-8 release from A549 cells after treatment for 24 h with the different ZnO samples and exposure conditions. The production of IL-8 showed significant differences between control groups (32, 49, and 61 pg/mL after exposure in DMEM, DMEM/5 % FBS, and DMEM/10 % FBS, respectively) (Fig. 8a). In the absence of serum, both nano- and micro-ZnO induced the greatest IL-8 expression at 20 μg/mL and then the expression decreased. Statistical analysis only shows significant differences in IL-8 production between nano- and micro-ZnO at 20 and 40 μg/mL, and shows significant differences between control and treated groups for nearly all dosages. In the presence of serum, the IL-8 expression in all treated groups was higher than in serum-free media. From one-way ANOVA analysis, significant IL-8 release from A549 cells treated with samples as compared to the control group can be observed (>20 μg/mL). However, inconsistent with the cell viability results, none of the groups had a significant effect on IL-8 production between nano- and micro-ZnO. Two factor ANOVA analyses revealed significant contrasts only related to serum conditions for all dosages after adjustment for particle size (*p* < 0.01), while no significant contrasts related to particle size after adjustment for serum conditions.

**Fig. 9:**
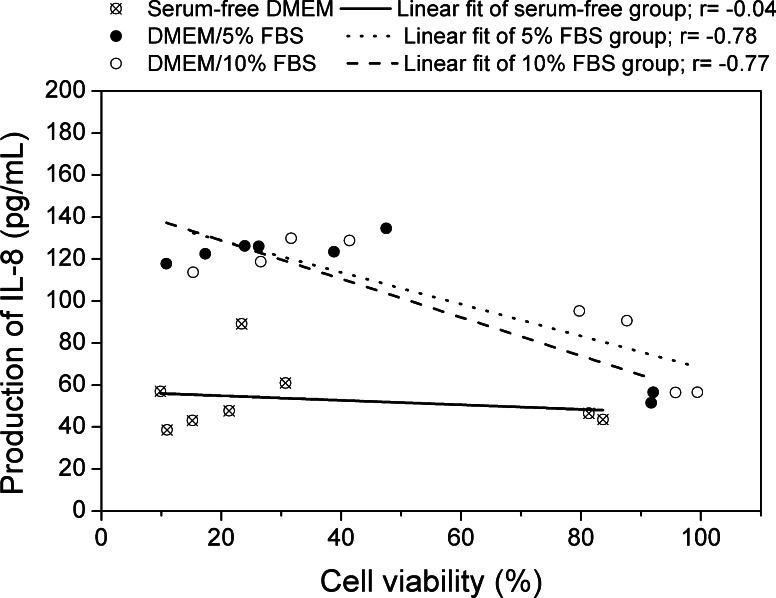
Correlations between mitochondrial activities (MTT) and inflammatory (IL-8) results of samples in A549 cells with various medium conditions. *r* correlation coefficients

To determine the association between cytotoxicity and inflammation responses as measured by MTT and IL-8 ELISA assay, linear correlation analyses were performed. Results are shown in Fig. 9. All samples within the same exposure condition were considered together, thus each condition had 8 units of data to make a correlation analysis. Significant inverse correlations were found between cell viability and production of IL-8 when A549 cells were treated in serum-containing media (in 5 % FBS, *r* = −0.78; in 10 % FBS, *r* = −0.77), whereas no correlation was observed as cells were treated in serum-free media (*r* = −0.04).

## Discussion

According to a predictive mathematical model from the International Commission on Radiological Protection (ICRP), particles having dimensions between 0.005 and 1 μm can deposit in the alveolar region of the human respiratory tract under nasal breathing conditions during rest (Bair 1994). In this study, the nano-ZnO particles that we tested had dimensions (theoretically) of less than 100 nm, although monitored data indicated that some NPs aggregated slightly to dimensions of less than 280 nm, due to the higher relative humidity in air (Abouzeid and Fuersten 1972). The size distribution of the micro-ZnO particles in air was also less than 1 μm. Therefore, both sets of particles that we used have the potential to deposit and have hazardous effects on alveolar epithelial cells. Before the particles come into contact with the cells, they would have to penetrate a surfactant-containing liquid layer (Kreyling and Geiser 2010). Such passage through this layer might lead to particle agglomeration, but the extent of agglomeration would depend on the surface charge of the particles and the presence of dispersants and adsorbed protein (Holgate 2010). Furthermore, the agglomeration of particles would also determine the cell uptake pathway (phagocytosis or pinocytosis) and the cytotoxicity (Casals et al. 2008). Therefore, to determine which medium components are most appropriate for evaluating NP cytotoxicity, it is important to calculate their size distributions in various media. When we dispersed our nano- and micro-ZnO particles in serum-free DMEM, they aggregated dramatically, presumably because of the high concentration of ionic salts in the medium (zeta potential close to zero) and also the presence of low amounts of amino acids, vitamins, and glucose (Allouni et al. 2009). We also observed decreases in the hydrodynamic sizes and zeta potentials after the addition of serum (at either 5 or 10 % in DMEM), presumably because of protein adsorption on the surfaces of particles (Allouni et al. 2009).

In addition to particle agglomeration, the rate of particle sedimentation is also relevant when evaluating the potential cytotoxicity of NPs (Limbach et al. 2005). The faster the particles deposit, the more likely they are to interact with the cells. In the serum-containing media, both the nano- and micro-ZnO formed more stable suspensions than they did in the serum-free medium, presumably because the albumin—the main component of serum protein—has a net negative charge in DMEM, allowing it to bind to positively charged ZnO through its carboxyl groups (RCOO^−^). The adsorption of these proteins on the surface of ZnO also induced steric stabilization. Micro-sized ZnO underwent faster sedimentation than did the nano-sized ZnO in all of the tested solutions, presumably because the weight of micro-ZnO aggregates was greater than that of the nano-ZnO aggregates. Recent research has made strides toward developing new technologies in NP dispersion for in vivo and in vitro studies (Bihari et al. 2008; Vippola et al. 2009). For example, the addition of dipalmitoylphosphatidylcholine (DPPC), the major component of lung surfactant, to serum-containing media provided a more stable, less-agglomerating suspension of TiO_2_ and carbon-based NPs (Vippola et al. 2009). In addition, the presence of DPPC also can modulate the oxidative stress response of human lung epithelial cells upon exposure to carbon nanomaterials (Herzog et al. 2009). To date, however, the most common dispersion agent used for in vitro studies has been either FBS or bovine serum albumin (Ji et al. 2010). The degree of metal ion release is another important toxicity resource. For example, Zn^2+^ is a toxic ion because it can inhibit mitochondrial respiration, leading to loss of intracellular adenosine triphosphate (Marin et al. 2000; Daniels et al. 2004). Xia et al. reported that the maximum total Zn^2+^ release from nano-ZnO in complete DMEM (DMEM/10 % FBS) was 225 μM (Xia et al. 2008). Deng et al. claimed that when less than 18.2 μg/mL of ZnO NPs was present, the cells were mainly exposed to aqueous Zn^2+^ (Deng et al. 2009). Song et al. used supernatants of ZnO particle suspension to treat mouse macrophage cells, and found significant correlations between Zn^2+^ concentration and cell viability results (Song et al. 2010). However, Park et al. evaluated negligible Zn^2+^ concentration from nano-ZnO in both neutral (PBS, pH 7.4) and acidic solvent (HCl, pH 4.5–5.0), and considered zinc ion release was not a critical factor in ZnO toxicity (Park et al. 2011). Ahamed et al. found that ZnO nano-rods released less than 10 μg/mL of Zn^2+^ at a particle concentration lower than 100 μg/mL, and that these levels of Zn^2+^ release were insufficient to induce cytotoxicity of A549 cells (Ahamed et al. 2011). In this study, we first observed similar degrees of Zn^2+^ release from both the nano- and micro-sized ZnO which was consistent with the results reported by Song et al. (2010). We also found that when dissolving 10 μg/mL of nano- and micro-ZnO in all conditions of media, around 8 μg/mL of Zn^2+^ was detected. By combining the bio-TEM observation (Supplementary Information), these results indicated that almost all ZnO particles were dissolved into zinc ion at this concentration. Our cytotoxicity results under this dosage had no significant differences compared with the control group in DMEM/10 % FBS media, while having significant differences in cytotoxicity between them in serum-free media. We examined the toxic effect of dissolved Zn^2+^ supernatants from 10 μg/mL of nano-ZnO and micro-ZnO in A549 cells and found that Zn^2+^ caused no apparent cytotoxicity in DMEM/10 % FBS media, while there was a slight decrease in cell viability under serum-free media, consistent with the cytotoxicity results of ZnO particles. Furthermore, we observed higher degrees of Zn^2+^ release in serum-containing media than serum-free media by dissolving 40 μg/mL of nano- and micro-ZnO. The cytotoxicity study for the supernatants received from 40 to 10 μg/mL of ZnO particles showed similar cytotoxicity in serum-free media, while still no apparent cytotoxicity was found from the supernatants in DMEM/10 % FBS media. These results suggest that dissolved Zn^2+^ in serum-free media made a greater impact on toxic responses of ZnO particles than that dissolved in serum-containing media, and some complex between the FBS protein and zinc ion occurred in serum-containing media. On the other hand, we found that ZnO in serum-containing media releases relatively more zinc ion than in serum-free media. The possible reason for the different levels of ZnO dissolution in water, serum-free and serum-containing media is discussed in Supplementary Information.

Cell growth is another important factor affected by the toxicity of NPs. We observed slower growth rate of A549 cells exposed to the serum-free medium, due to the absence of growth nutrients and factors. Even if there were an equal cell density prior to the exposure step, cells undergoing a slower growth rate would lead to a lower cell density. Heng et al. demonstrated that a sparse monolayer cell density was less resistant to the cytotoxicity of nano-ZnO compared to a higher cell density for four different cell lines (Heng et al. 2011). A higher relative dosage of particles to cells, attributed to lower cell density, might induce a more serious cytotoxicity. Furthermore, the starved cells were incubated in a nutrient-absent medium, which might trend more toward the uptake of ZnO particles; but severe particle aggregation in a serum-free medium might vary the uptake mechanism and lower the uptake. Thus, the uptake level of ZnO particles in time and space resolved should be further studied. The strategy developed by Shapero et al. could be utilized (Shapero et al. 2011).

The effect of protein adsorption on the cytotoxicity of NPs has been noted in several reports (Horie et al. 2009; Ge et al. 2011; Hu et al. 2011). For example, the presence of serum in a medium can have a protective effect on the toxicity of single-walled carbon nanotubes and graphene oxide (Ge et al. 2011; Hu et al. 2011). One report concluded that nano-ZnO requires cell contact to exhibit its cytotoxicity (Moos et al. 2010). Based on the bio-TEM results, the production of nano-bio-complex in DMEM/10 % FBS might cause less cell–particle contact, thereby decreasing the cytotoxicity. In addition, we should not neglect considering possible different components of protein corona in the absence or presence of serum effects on particle–cell interaction (Cedervall et al. 2007; Lynch and Dawson 2008; Lynch et al. 2009; Nel et al. 2009; Walkey and Chan 2012), and possible protein conformation change/aggregation induced by NPs that destroy protein activity (Prapainop et al. 2012; Walkey and Chan 2012).

We observed size-dependent toxicity of nano- and micro-ZnO in both media, with and without serum at dosages ≥ 20 μg/mL. Similar results have been reported previously. Moos et al. noted that nano-sized ZnO was more toxic than micrometer-sized ZnO toward human colon cancer cells (RKO) (Moos et al. 2010). Nair et al. revealed that the toxicity of ZnO NPs was greater than that of ZnO microparticles toward human osteoblast cancer cells (MG-63) (Nair et al. 2009). Furthermore, Hanley et al. also reported significantly greater cytotoxicity of 4-nm ZnO NPs relative to 20-nm ZnO NPs (Hanley et al. 2009). In contrast, Deng et al. found no size-dependent toxicity of 10-, 30-, 60-, and 200-nm ZnO NPs for mouse neural stem cells (Deng et al. 2009). Yuan et al. reported that the cytotoxicity was independent of the size of nano-ZnO (20, 30, and 40 nm) to human embryonic lung fibroblasts (Yuan et al. 2010). The reason for an absence of a size-dependent effect might be the dissolution of Zn^2+^ (Deng et al. 2009), or the size of ZnO did not exceed a certain critical size (Song et al. 2010). In this present study, we inferred that for concentrations of ZnO in the media ≤10 μg/mL, there was no size-dependent toxicity due to all particles being dissolved into Zn^2+^. When the dosage of ZnO particles was ≥ 20 μg/mL, the degree of Zn^2+^ release increased in serum-containing media. We also noted a similar degree of Zn^2+^ release from the nano- and micro-ZnO under otherwise identical conditions. In addition, Zn^2+^ release from ZnO particles exhibited toxic response to cells only in serum-free media (10–40 μg/mL of ZnO supernatants). These results suggest that although the presence of Zn^2+^ in serum-containing or serum-free media did not affect, or slightly affected, the cytotoxicity of ZnO, the non-dissolved remnants of the NPs play a major role in influencing their cytotoxicity. In our previous study, we found that smaller-sized nano-ZnO (of both rod and spherical shapes), with a smaller primary size, formed a relatively smaller secondary size in serum-free medium, thereby resulting in more cytotoxicity relative to larger-sized nano-ZnO (Hsiao and Huang 2011a). In this present study, we confirmed ZnO particles having a smaller primary size aggregated to a relatively smaller secondary size in all of the tested media. In the same mass dosage, larger particle numbers and surface areas for smaller particle sizes resulted in size-dependent cytotoxicity. It also concluded that mass dosimetry is appropriate to compare the toxicity between these two size of material due to there were totally different components of zinc at various dosages. However, if ZnO dissolves very quickly, e.g., ≤10 μg/mL, then dissolution rate dominates over size dependency and no cytotoxicity differences were observed. Though the Zn^2+^ ions within the extracellular environment contributed little to cytotoxicity of higher concentrations of ZnO particulates, it did not mean that possible intracellular Zn^2+^, which dissolved in acidic conditions within the lysosomal and alveolar compartments of cells after ZnO particle uptake, would also show little effect, because the mechanisms are totally different.

The opposite dose-dependent effect on the two sizes of ZnO could be observed between mass-based and SSA-based dosimetries. The results correspond to the Lin’s research (Lin et al. 2009). Our data further revealed that there was no correlation between particle surface area and cytotoxicity as in serum-containing media, while in serum-free exposure the surface area dependent toxicity could be observed. Many reasons include increase of ZnO dissolution, lower sedimentation rate and serum-coated particles in serum-containing media all may explain our results. They all decrease the toxic contribution from the surface of particles. These findings suggested that it’s inappropriate to use surface area dosimetry in studying the cytotoxicity of ZnO in serum-containing media because the “real” surface area of the particles contacting the cells determines the toxicity (Hsiao and Huang 2011a).

Cronholm et al. found less particle agglomeration and increased Cu release in serum-containing medium (Cronholm et al. 2011), while having no clear difference in toxicity to A549 cells. This study tried to figure out and discuss which factors might affect the toxicity difference between serum and serum-free media. The possible factors to be considered include physicochemical properties and cellular behaviors. Table 2 summarizes possible influencing factors that contributed to the cytotoxicity of nano-ZnO in various media. For the NPs in the serum-free medium, large aggregates of particles lead to rapid sedimentation. Thus, larger parts of particles have more opportunities to directly contact with cells in the absence of protein protection. Collaborating with a low cell-growth rate, these factors contribute to high dosage-per-cell ratio. Though less Zn^2+^ release was observed in the serum-free medium, it was more potentially toxic than that in serum-containing media. On the other hand, the physicochemical properties of nano-ZnO were similar both in 5 and 10 % FBS-containing media, while cytotoxicity differences still existed. This indicated that cell growth rate dominated this difference within the serum-containing media.

**Table 2:** Possible factors affecting nano-ZnO cytotoxicity in various media

Factor/medium	Serum-free DMEM	DMEM/5 % FBS	DMEM/10 % FBS
Secondary size	Large	Small	Small
Sedimentation rate	Fast	Slow	Slow
Zinc ion release	Low	–	High
Protein protection	No	Yes	Yes
Cell growth rate	Slow	Medium	Fast
Resultant toxicity	High	Medium	Low

IL-8 is an important chemokine to mediate pulmonary inflammation, and also a good marker to evaluate an intermediate level (Tier 2) of oxidative stress on in vitro and in vivo studies (Nel et al. 2006). In vitro studies have also demonstrated that NPs can up-regulate IL-8 in human lung epithelial cells. Baktur et al. demonstrated that IL-8 expression is enhanced in the presence of serum when carbon nanotubes (SWCNTs) were exposed to A549 cells even if a lower cytotoxicity was observed (Baktur et al. 2011). This is because more SWCNTs penetrated into cells in the presence of serum. Our study also found the same IL-8 results for both ZnO particles. Both the uptake mechanism and level might affect the expression, but more biological information was required to explain. Interestingly, the IL-8 expression in three control groups showed high correlation with its corresponding OD values of MTT assay (*r* = 0.95). This suggested that more cell numbers for both control and treated groups in serum-containing medium contributed to a higher expression. Actually, if we changed the *y*-axis expression [production of IL-8 (pg/mL)] in Fig. 8b to a percentage of IL-8 release to control (%) as a substitute to compare the releasing folder between each case, nano-ZnO in the serum-free medium even induced the highest fold (approximately 2.8 times) for the IL-8 at 20 μg/mL than that in the serum-containing medium at all dosages. In addition, size-dependent inflammation response was observed only in groups of serum-free media. These results suggested that cells were more sensitive to xenobiotics in a nutrient-deficient state. On the other hand, it showed high association between cell viability and IL-8 expression data in serum-containing media, which is consistent with some previous studies (De Berardis et al. 2010; Perrone et al. 2010). However, the correlation was not found in serum-free media, implying that some signaling pathways might alter under this condition.

In general, in vitro studies are performed using serum-free media to avoid complicated particle–protein interactions as, for example, shown by Casey et al. who demonstrated that FBS interacts with carbon nanotubes (Casey et al. 2007), or in other cases to avoid interference in biological assays [e.g., from lactate dehydrogenase (Hollaar and Vanderlaarse 1979)]. In our present study, however, serious agglomeration of the NPs in the serum-free medium may have led to loss of their original surface properties and rapid particle–cell interaction. Furthermore, in the absence of nutrients and proteins, cultures might experience increased cell stress, increased phagocytosis (Walker et al. 1991), and altered transcription behavior for protein production (Jones and Grainger 2009). Although we did not observe a cytokine adsorption effect for ZnO NPs in our previous studies, this effect does occur with negatively charged NPs [e.g., TiO_2_ (Hsiao and Huang 2011b)]. These implications might lead to errors in determining “real world” toxicity. After all, no in vivo system features an absence of serum protein. In the absence of any special reasons, we recommend the use of a serum-containing medium when evaluating the cytotoxicity of NPs. Finally, in selecting stabilizing agents for in vitro testing, it is important to consider not only from biological aspects (e.g., bioavailability and real situation of exposure), but also from NPs aspects (e.g., additional dissolution and surface charge alternation).

## Conclusion

In this study, we have demonstrated that the degrees of ZnO cytotoxicity are different when the particles are exposed to either serum-containing or serum-free media. In serum-free media, higher ZnO toxicity ensues, not only because of large aggregates of particles, rapid sedimentation of suspension, larger parts of particles directly contacting with cells in absence of protein protection, and a lower cell growth rate, all contributing to high dosage-per-cell ratio, but also because Zn^2+^ release from ZnO particles contribute to cytotoxicity. In serum-containing media, even though two kinds of ZnO featured a smaller particle size than that in serum-free media, the faster cell growth rate, formation of a stable suspension, lower cytotoxicity response to Zn^2+^, and serum protection all led to lower cytotoxicity. Moreover, we also demonstrated that no matter which medium, extracellular Zn^2+^ release did not contribute as a main factor in cytotoxicity of nano-ZnO, suggesting that the non-dissolved remnants of the ZnO particles must contribute to their cytotoxicity; thereby explaining the observed size-dependent toxicity at higher exposure concentration. Finally, since growth rate, transcription behavior of cells, and physicochemical properties of ZnO particles all might be altered in a serum-free exposure condition, the addition of FBS to the media might appear to be appropriate when assessing the cytotoxicity of NPs.

## Electronic supplementary material

Below is the link to the electronic supplementary material.

Supplementary material 1 (DOC 4902 kb)
