# Supplementary material for: Effects of serum on cytotoxicity of nano- and micro-sized ZnO particles
Source: J Nanopart Res. 2013 Aug 27;15(9):1829. doi: 10.1007/s11051-013-1829-5 (PMC3782657; doi:10.1007/s11051-013-1829-5)
Supplement: Supplementary file 1 — Supplementary material 1 (DOC 4902 kb) [file 11051_2013_1829_MOESM1_ESM.doc]

**Supplementary Information**

**Effects of serum on cytotoxicity of nano- and micro-sized ZnO particles**

***Journal of Nanoparticle Research***

I-Lun Hsiao and Yuh-Jeen Huang*

Department of Biomedical Engineering and Environmental Sciences, National Tsing Hua University, Hsinchu, Taiwan, R.O.C.

Email: yjhuang@mx.nthu.edu.tw

**1. Morphologies of nano-ZnO in DMEM and DMEM/10%FBS media**

In order to further confirm the dissolution of nano-ZnO in DMEM and DMEM/10%FBS, we used bio-TEM to check it.

***Method***

Nano-ZnO particles from a stock solution (2 mg/mL) were diluted to 40 and 10 μg /mL with serum-free DMEM and DMEM/10%FBS, then incubated for 24 h at 37 °C. Suspensions were mixed homogeneously. Aliquots (4 μL) of the solutions were deposited on 200-mesh carbon-formvar Cu grids. Finally, the grids were treated in a vacuum oven for 24 h at 70 °C. The morphology of nano-ZnO was examined using Bio-TEM (Hitachi HT7700), with 100 kV acceleration voltage.

***Results and discussion***

In the TEM image, nano-ZnO suspension (40 μg/mL) in DMEM maintained its original size and shape (Fig.S1). However, by enlarging the image, we observed that the surface of nanoparticles became rough, which suggested some dissolution occurred. When nano-ZnO was suspended at 10 μg/mL in DMEM, we could not find any agglomerates of nano-ZnO in the TEM image (Fig. S2). Only a little debris could be observed, suggesting in this case that almost all ZnO particles dissolve into serum-free DMEM media.

For nano-ZnO suspended into DMEM/10%FBS at 40 μg/mL, we found many uniformed and spherical particles (50~150 nm in size), while no originally shaped particles (60 nm, irregular shape) could be observed (Fig. S3). Xu et al. have done the same experiments as those in our work . From HR-TEM images, they found ZnO nanoparticles were surrounded by large amounts of bio-complex. However, the size of these ZnO nanoparticles were mostly smaller than the original one (<10 nm versus 60 nm). Thus, we supposed that our nano-ZnO dissolved into very tiny particles and generated a nano-bio-complex in DMEM/10%FBS at 40 μg/mL. When 10 μg/mL of nano-ZnO was suspended in DMEM/10%FBS, a similar morphology as at 40 μg/mL was observed (Fig. S4). Due to formation of aggregates/agglomerates of proteins in a serum-containing medium could be observed even when there was no nano-ZnO in it, it is hard to identify whether the nano-ZnO at 10 μg/mL totally dissolved or not in DMEM/10%FBS by TEM.


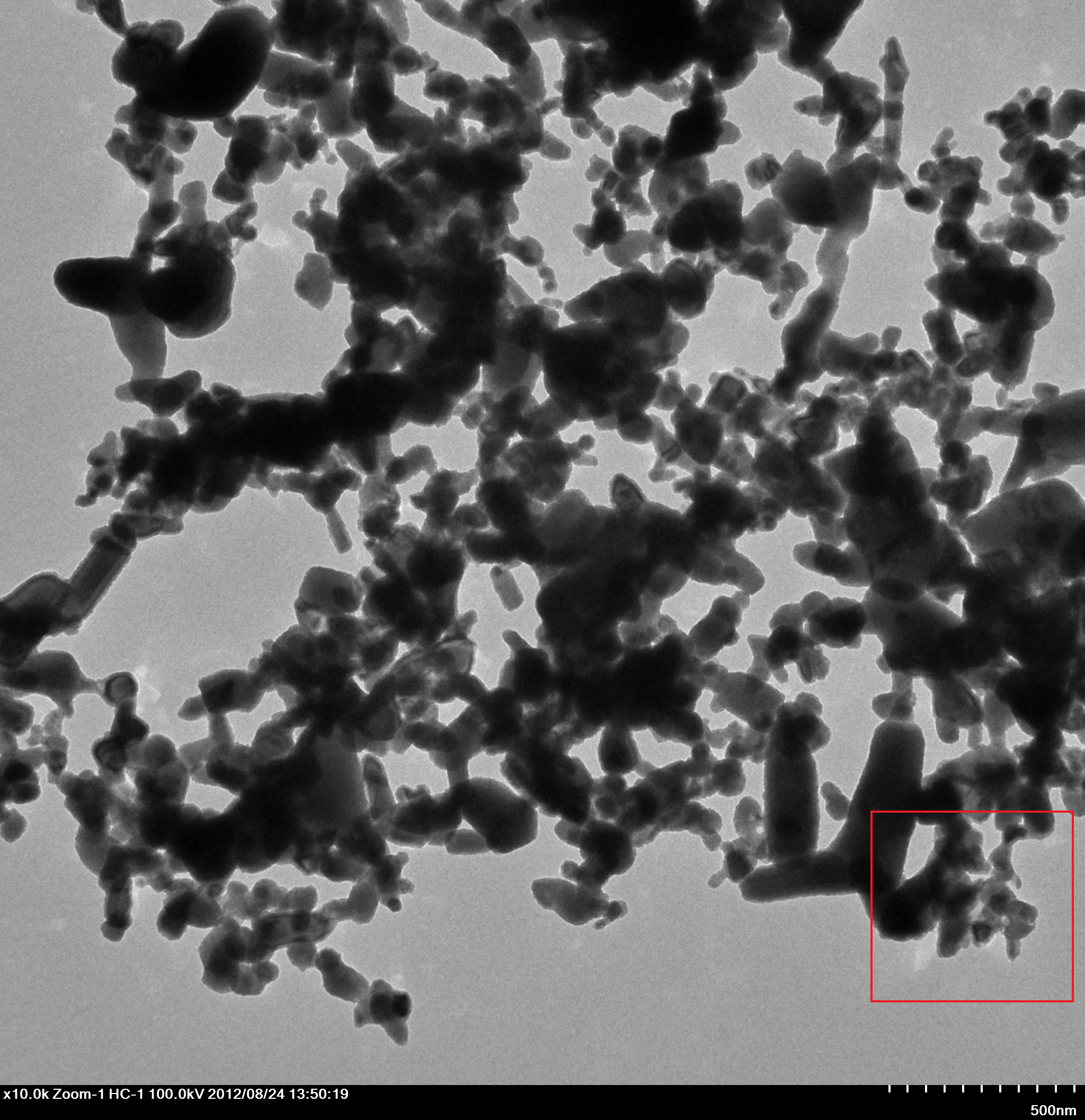

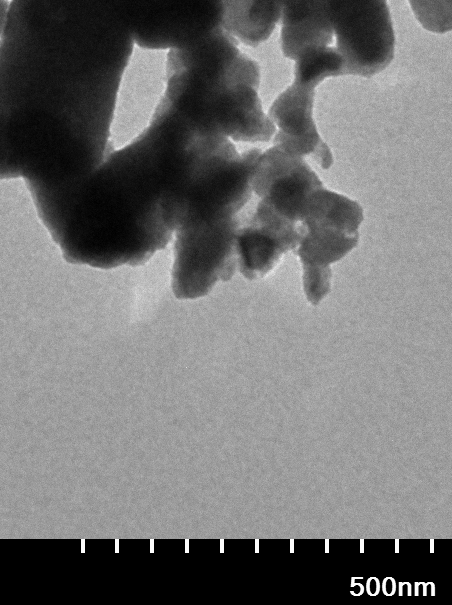


**A**

**B**

**Figure S1.** Nano-ZnO suspended in DMEM medium (40 μg/mL) (A); Enlarged figure (B)


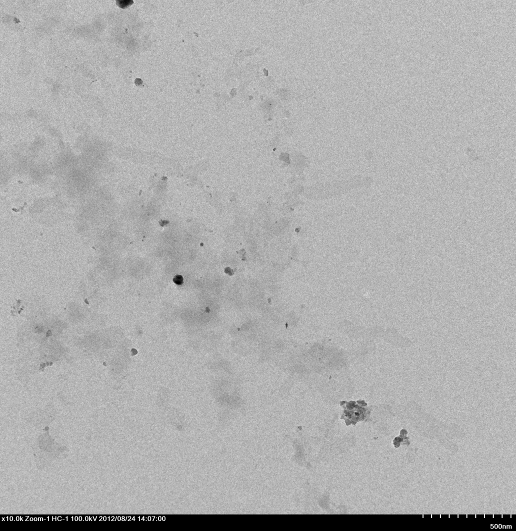


**Figure S2.** Nano-ZnO suspended in DMEM medium (10 μg/mL)


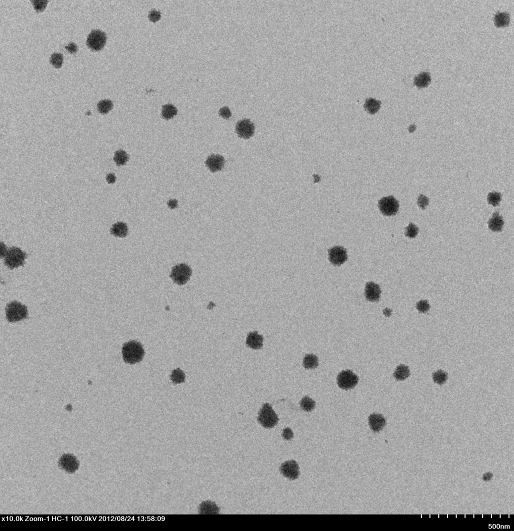


**Figure S3.** Nano-ZnO suspended in DMEM/10%FBS medium (40 μg/mL)


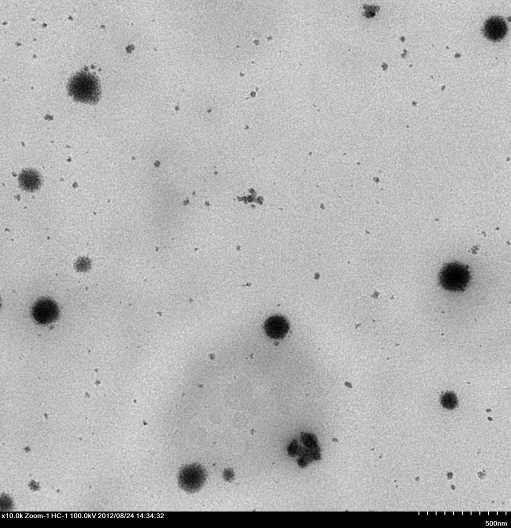


**Figure S4.** Nano-ZnO suspended in DMEM/10%FBS medium (10 μg/mL)

**2. Discussion of ZnO dissolution**

In general, bulk ZnO has low dissolution in water (Ksp = 1.6 × 10^-17^) (corresponding to 0.16 ppm ZnO dissolved). The following reactions are important in water at pH 7.5:

ZnO _(s)_ + H_2_O _(l)_ → Zn(OH)_2_ _(s)_

Zn(OH)_2_ _(s)_ → Zn(OH)^+^ _(aq)_ + OH^-^ _(aq)_

Zn(OH)^+^ _(aq)_ → Zn^2+^ _(aq)_ + OH^-^ _(aq)_

Overall reaction: ZnO + H_2_O _(l)_ →Zn^2+^ _(aq)_ + 2OH^-^ _(aq)_

The pH value of the medium DMEM ranged from 7.0~7.5, but we found that 10 μg/mL of ZnO suspension released about 8 μg/mL Zn^2+^ in DMEM. There are two possible reasons to explain the increased ZnO dissolution in cell medium. High concentration of ionic salts in medium, such as NaCl, increases ionic strength and promotes dissolution, which can be explained by Debye–Hückel theory. Moreover, Ai et al. also mention that amino acid (e.g., Glycin) and zinc ion could form stable chelates in a biological system . This reaction may also increase the dissolution of ZnO nanoparticles in medium.

When the fetal bovine serum (FBS) was added in DMEM medium, more zinc ion released from ZnO suspension. The reason might be because the serum protein have many carboxyl groups (RCOO^−^) and the carboxyl-groups-rich compound－citrate. These functional groups adsorb to the surface of ZnO. Based on the study of Grassian’s group, carboxyl group-containing ligand citric acid can form a strong complex between metal cation and the ligand . This leads to a weak metal-oxide bond and then results in a higher degree of ZnO dissolution. We supposed serum protein also could undergo this mechanism.

We also found that there were no significant differences in levels of ZnO dissolution between nano- and micro-ZnO in both cell media. However, according to the Ostwald-Friedrich equation, solubility will increase exponentially with decreasing particle size.

Grassian’s group claimed that size-dependent solubility is derived from nanoparticles having more edges, kinks and high defect site density as “hot spots” of dissolution. However, the particle size dependence of ZnO was not as prevalent in the presence of ligands (e.g. citric acid). When ligands exist, the hot spots are created on the terraces of particles, rather than edges or kinks . This causes the same extent of dissolution in both nano and bulk materials, masking size-dependent dissolution.

Ostwald-Friedrich equation:


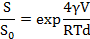


where S is the solubility (mol/kg) of a spherical particle with diameter d (m), S_0_(bulk) is the solubility of the bulk material, γ is the surface free energy (mJ/m^2^), R is the gas constant (mJ/mol K), V is the molecular volume (m^3^/mol), and T is the temperature (K).

References:

Ai HQ, Bu YX, Han KL (2003) Glycine-Zn^+^/Zn^2+^ and their hydrates: On the number of water molecules necessary to stabilize the switterionic glycine-Zn^+^/Zn^2+^ over the nonzwitterionic ones. J Chem Phys 118 (24):10973-10985. doi: 10.1063/1.1575192

Mudunkotuwa IA, Rupasinghe T, Wu CM, Grassian VH (2012) Dissolution of ZnO nanoparticles at circumneutral pH: a study of size effects in the presence and absence of citric acid. Langmuir 28 (1):396-403. doi: 10.1021/la203542x

Xu MS, Li J, Iwai H, Mei QS, Fujita D, Su HX, Chen HZ, Hanagata N (2012) Formation of Nano-Bio-Complex as Nanomaterials Dispersed in a Biological Solution for Understanding Nanobiological Interactions. Sci Rep-Uk 2. doi: 10.1038/Srep00406
